# 4-[(3-Oxo-1,3-dihydro-2-benzofuran-1-yl)amino]­benzoic acid

**DOI:** 10.1107/S1600536810048695

**Published:** 2010-11-30

**Authors:** Chao-Jun Du

**Affiliations:** a School of Biology and Chemical Engineering, Nanyang Institute of Technology, Nanyang 473004, People’s Republic of China

## Abstract

In the title compound, C_15_H_11_NO_4_, the dihedral angle formed by the benzene ring and the essentially planar 2-benzofuran ring system is 55.93 (3)°. In the crystal, inter­molecular O—H⋯O hydrogen bonds link pairs of mol­ecules, generating centrosymmetric *R*_2_^2^(8) ring motifs. These dimeric units are connected via N—H⋯O hydrogen bonds, forming *C*(6) chains along [100].

## Related literature

For the structure of 2-(3-oxo-1,3-dihydro­isobenzofuran-1-yl­amino)­benzoic acid, see: Odabaşoğlu & Büyükgüngör (2008) ▶. For the structure of 3-(3-oxo-1,3-dihydro­isobenzofuran-1-yl­amino)­benzoic acid, see: Li *et al.* (2009 ▶). For hydrogen-bond motifs, see: Bernstein *et al.* (1995 ▶).
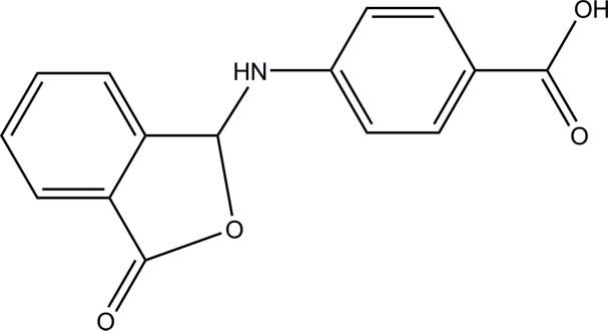


## Experimental

### 

#### Crystal data

C_15_H_11_NO_4_
*M* *_r_* = 269.25
Triclinic, 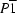

*a* = 5.9727 (18) Å
*b* = 6.987 (2) Å
*c* = 15.451 (5) Å
α = 78.135 (3)°
β = 87.217 (3)°
γ = 77.804 (3)°
*V* = 616.8 (3) Å^3^
*Z* = 2
Mo *K*α radiation
μ = 0.11 mm^−1^
*T* = 296 K
0.21 × 0.16 × 0.11 mm

#### Data collection

Bruker APEXII CCD diffractometer
Absorption correction: multi-scan (*SADABS*; Sheldrick, 1996 ▶) *T*_min_ = 0.98, *T*_max_ = 0.988
5940 measured reflections
2213 independent reflections
1971 reflections with *I* > 2σ(*I*)
*R*_int_ = 0.015

#### Refinement

*R*[*F* ^2^ > 2σ(*F* ^2^)] = 0.035
*wR*(*F* ^2^) = 0.101
*S* = 1.05
2213 reflections
182 parameters
H-atom parameters constrained
Δρ_max_ = 0.21 e Å^−3^
Δρ_min_ = −0.26 e Å^−3^

### 

Data collection: *APEX2* (Bruker, 2004 ▶); cell refinement: *SAINT* (Bruker, 2004 ▶); data reduction: *SAINT*; program(s) used to solve structure: *SHELXS97* (Sheldrick, 2008 ▶); program(s) used to refine structure: *SHELXL97* (Sheldrick, 2008 ▶); molecular graphics: *SHELXTL* (Sheldrick, 2008 ▶); software used to prepare material for publication: *SHELXTL*.

## Supplementary Material

Crystal structure: contains datablocks I, global. DOI: 10.1107/S1600536810048695/lh5161sup1.cif

Structure factors: contains datablocks I. DOI: 10.1107/S1600536810048695/lh5161Isup2.hkl

Additional supplementary materials:  crystallographic information; 3D view; checkCIF report

## Figures and Tables

**Table 1:** Hydrogen-bond geometry (Å, °)

*D*—H⋯*A*	*D*—H	H⋯*A*	*D*⋯*A*	*D*—H⋯*A*
N1—H1⋯O4^i^	0.88	2.11	2.9802 (17)	168
O1—H1*A*⋯O2^ii^	0.86	1.79	2.6438 (16)	175

Symmetry codes: (i) 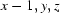; (ii) 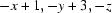.

**Fig. 1.**
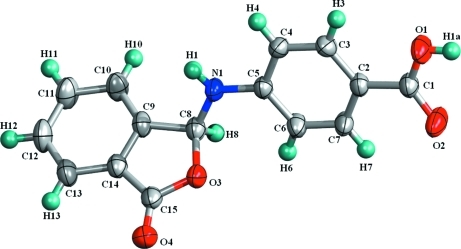
The molecular structure of the title compound with 50% probability displacement ellipsoids.

**Fig. 2.**
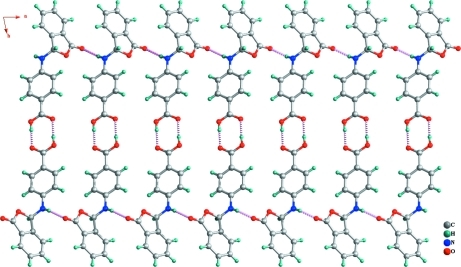
Part of the crystal structure with hydrogen bonds shown as dashed lines.

## supplementary crystallographic information

### Comment

The molecular structure of the title compound is shown in Fig. 1. The dihedral angle formed by the benzene ring and the essentially planar isobenzofuran ring system is 55.93 (3)°. In the crystal, intermolecular O—H···O hydrogen bonds link pairs of molecules, generating centrosymmetric *R*_2_^2^(8) ring motifs. These dimeric units are connected via N—H···O hydrogen bonds forming C(6) chains along [100] (see Fig. 2). The crystal structures of 2-(3-oxo-1,3-dihydroisobenzofuran-1-ylamino)benzoic acid (Odabaşoğlu & Büyükgüngör, 2008) and 3-(3-oxo-1,3-dihydroisobenzofuran-1-ylamino)benzoic (Li *et al.*(2009) have been published previously.

### Experimental

4-aminobenzoic acid (5.00 mmol) and 2-formylbenzoic acid (5.00 mmol) were added to an ethanol (35 ml) and DMF (15 ml) mix. The mixture was stirred at 353 K for 5 h. The resulting clear solution was evaporated under vacuum. The product was crystallized from a solution of DMF/methanol(1:1) yielding the title compound. Anal. yield: *ca* 98.6%. Single crystals suitable for X-ray analysis were obtained within one week by slow evaporation of a DMF/methanol (1:3) solution of the title compound.

### Refinement

All H atoms were placed in idealized positions (C—H = 0.93 or 0.98 Å, N—H = 0.88 Å and O—H = 0.86 Å), and constrained to ride on the atom to which they are bonded, and were included in the refinement in the riding-model approximation. *U*_iso_(H)  values were set equal to 1.5*U*_eq_(parent atom) for carboxyl and the secondary amine H atom and to 1.2*U*_eq_(parent atom) for all other H atoms.

### Crystal data

**Table:**

C_15_H_11_NO_4_	*Z* = 2
*M**_r_* = 269.25	*F*(000) = 280
Triclinic, *P*1	*D*_x_ = 1.450 Mg m^−^^3^
Hall symbol: -P 1	Mo *K*α radiation, λ = 0.71073 Å
*a* = 5.9727 (18) Å	Cell parameters from 3521 reflections
*b* = 6.987 (2) Å	θ = 2.7–27.8°
*c* = 15.451 (5) Å	µ = 0.11 mm^−^^1^
α = 78.135 (3)°	*T* = 296 K
β = 87.217 (3)°	Block, colorless
γ = 77.804 (3)°	0.21 × 0.16 × 0.11 mm
*V* = 616.8 (3) Å^3^	

### Data collection

**Table:**

Bruker APEXII CCD diffractometer	2213 independent reflections
Radiation source: fine-focus sealed tube	1971 reflections with *I* > 2σ(*I*)
graphite	*R*_int_ = 0.015
φ and ω scans	θ_max_ = 25.2°, θ_min_ = 2.7°
Absorption correction: multi-scan (*SADABS*; Sheldrick, 1996)	*h* = −7→7
*T*_min_ = 0.98, *T*_max_ = 0.988	*k* = −8→8
5940 measured reflections	*l* = −18→18

### Refinement

**Table:**

Refinement on *F*^2^	Primary atom site location: structure-invariant direct methods
Least-squares matrix: full	Secondary atom site location: difference Fourier map
*R*[*F*^2^ > 2σ(*F*^2^)] = 0.035	Hydrogen site location: inferred from neighbouring sites
*wR*(*F*^2^) = 0.101	H-atom parameters constrained
*S* = 1.05	*w* = 1/[σ^2^(*F*_o_^2^) + (0.0516*P*)^2^ + 0.1431*P*] where *P* = (*F*_o_^2^ + 2*F*_c_^2^)/3
2213 reflections	(Δ/σ)_max_ < 0.001
182 parameters	Δρ_max_ = 0.21 e Å^−^^3^
0 restraints	Δρ_min_ = −0.26 e Å^−^^3^

### Special details

**Table:**

Geometry. All e.s.d.'s (except the e.s.d. in the dihedral angle between two l.s. planes) are estimated using the full covariance matrix. The cell e.s.d.'s are taken into account individually in the estimation of e.s.d.'s in distances, angles and torsion angles; correlations between e.s.d.'s in cell parameters are only used when they are defined by crystal symmetry. An approximate (isotropic) treatment of cell e.s.d.'s is used for estimating e.s.d.'s involving l.s. planes.
Refinement. Refinement of *F*^2^ against ALL reflections. The weighted *R*-factor *wR* and goodness of fit *S* are based on *F*^2^, conventional *R*-factors *R* are based on *F*, with *F* set to zero for negative *F*^2^. The threshold expression of *F*^2^ > σ(*F*^2^) is used only for calculating *R*-factors(gt) *etc*. and is not relevant to the choice of reflections for refinement. *R*-factors based on *F*^2^ are statistically about twice as large as those based on *F*, and *R*- factors based on ALL data will be even larger.

### Fractional atomic coordinates and isotropic or equivalent isotropic displacement parameters (Å^2^)

**Table:**

	*x*	*y*	*z*	*U*_iso_*/*U*_eq_	
C1	0.5514 (2)	1.2253 (2)	0.06142 (9)	0.0419 (3)	
C2	0.5971 (2)	1.0142 (2)	0.10724 (9)	0.0380 (3)	
C3	0.4306 (2)	0.9320 (2)	0.15965 (10)	0.0413 (3)	
H3	0.2840	1.0090	0.1624	0.050*	
C4	0.4797 (2)	0.7390 (2)	0.20729 (10)	0.0409 (3)	
H4	0.3668	0.6872	0.2422	0.049*	
C5	0.6981 (2)	0.62056 (19)	0.20341 (9)	0.0363 (3)	
C6	0.8628 (2)	0.6997 (2)	0.14808 (9)	0.0440 (4)	
H6	1.0072	0.6210	0.1428	0.053*	
C7	0.8119 (3)	0.8932 (2)	0.10154 (9)	0.0443 (4)	
H7	0.9235	0.9443	0.0655	0.053*	
C8	0.9645 (2)	0.3035 (2)	0.25671 (9)	0.0403 (3)	
H8	1.0056	0.2839	0.1965	0.048*	
C9	0.9832 (2)	0.10315 (19)	0.31764 (9)	0.0389 (3)	
C10	0.8538 (3)	−0.0406 (2)	0.32249 (10)	0.0498 (4)	
H10	0.7294	−0.0204	0.2854	0.060*	
C11	0.9156 (3)	−0.2158 (2)	0.38446 (11)	0.0581 (4)	
H11	0.8300	−0.3144	0.3894	0.070*	
C12	1.1024 (3)	−0.2478 (2)	0.43950 (11)	0.0573 (4)	
H12	1.1423	−0.3685	0.4795	0.069*	
C13	1.2293 (3)	−0.1033 (2)	0.43565 (10)	0.0495 (4)	
H13	1.3534	−0.1229	0.4729	0.059*	
C14	1.1648 (2)	0.0728 (2)	0.37402 (9)	0.0397 (3)	
C15	1.2613 (2)	0.2526 (2)	0.35724 (10)	0.0433 (3)	
N1	0.7473 (2)	0.43002 (16)	0.25574 (8)	0.0417 (3)	
H1	0.6533	0.4023	0.3003	0.063*	
H1A	0.3357	1.4504	0.0381	0.063*	
O1	0.35440 (19)	1.33180 (15)	0.06907 (8)	0.0583 (3)	
O2	0.7119 (2)	1.29313 (16)	0.01765 (8)	0.0654 (4)	
O3	1.14390 (17)	0.38706 (15)	0.29107 (7)	0.0478 (3)	
O4	1.41808 (19)	0.28973 (18)	0.39302 (8)	0.0595 (3)	

### Atomic displacement parameters (Å^2^)

**Table:**

	*U*^11^	*U*^22^	*U*^33^	*U*^12^	*U*^13^	*U*^23^
C1	0.0449 (8)	0.0338 (7)	0.0402 (7)	−0.0024 (6)	0.0006 (6)	0.0019 (6)
C2	0.0421 (7)	0.0327 (7)	0.0344 (7)	−0.0034 (6)	−0.0009 (6)	0.0000 (5)
C3	0.0338 (7)	0.0347 (7)	0.0503 (8)	−0.0015 (6)	−0.0016 (6)	−0.0019 (6)
C4	0.0346 (7)	0.0359 (7)	0.0488 (8)	−0.0088 (6)	0.0014 (6)	0.0006 (6)
C5	0.0402 (7)	0.0292 (7)	0.0368 (7)	−0.0060 (5)	−0.0035 (5)	−0.0006 (5)
C6	0.0398 (8)	0.0375 (8)	0.0440 (8)	0.0031 (6)	0.0058 (6)	0.0033 (6)
C7	0.0433 (8)	0.0399 (8)	0.0399 (7)	−0.0030 (6)	0.0079 (6)	0.0068 (6)
C8	0.0446 (8)	0.0325 (7)	0.0387 (7)	−0.0034 (6)	−0.0005 (6)	−0.0004 (6)
C9	0.0472 (8)	0.0300 (7)	0.0355 (7)	−0.0018 (6)	0.0008 (6)	−0.0040 (5)
C10	0.0656 (10)	0.0345 (7)	0.0491 (8)	−0.0105 (7)	−0.0088 (7)	−0.0058 (6)
C11	0.0833 (12)	0.0326 (8)	0.0593 (10)	−0.0185 (8)	−0.0057 (9)	−0.0037 (7)
C12	0.0818 (12)	0.0324 (8)	0.0495 (9)	−0.0058 (8)	−0.0033 (8)	0.0052 (6)
C13	0.0547 (9)	0.0407 (8)	0.0443 (8)	−0.0007 (7)	−0.0043 (7)	0.0027 (6)
C14	0.0408 (7)	0.0351 (7)	0.0377 (7)	−0.0013 (6)	0.0033 (6)	−0.0025 (6)
C15	0.0363 (7)	0.0413 (8)	0.0451 (8)	−0.0034 (6)	0.0035 (6)	0.0025 (6)
N1	0.0402 (6)	0.0314 (6)	0.0459 (7)	−0.0041 (5)	0.0018 (5)	0.0055 (5)
O1	0.0537 (7)	0.0341 (6)	0.0706 (8)	0.0054 (5)	0.0083 (6)	0.0104 (5)
O2	0.0602 (7)	0.0409 (6)	0.0777 (8)	−0.0019 (5)	0.0204 (6)	0.0142 (6)
O3	0.0423 (6)	0.0378 (5)	0.0561 (6)	−0.0092 (4)	−0.0037 (5)	0.0091 (4)
O4	0.0455 (6)	0.0632 (7)	0.0657 (7)	−0.0194 (5)	−0.0085 (5)	0.0068 (6)

### Geometric parameters (Å, °)

**Table:**

C1—O2	1.2640 (18)	C8—H8	0.9800
C1—O1	1.2670 (17)	C9—C14	1.378 (2)
C1—C2	1.4736 (19)	C9—C10	1.379 (2)
C2—C7	1.389 (2)	C10—C11	1.383 (2)
C2—C3	1.395 (2)	C10—H10	0.9300
C3—C4	1.3755 (19)	C11—C12	1.388 (3)
C3—H3	0.9300	C11—H11	0.9300
C4—C5	1.395 (2)	C12—C13	1.375 (2)
C4—H4	0.9300	C12—H12	0.9300
C5—N1	1.3884 (17)	C13—C14	1.387 (2)
C5—C6	1.399 (2)	C13—H13	0.9300
C6—C7	1.3729 (19)	C14—C15	1.462 (2)
C6—H6	0.9300	C15—O4	1.2086 (18)
C7—H7	0.9300	C15—O3	1.3476 (17)
C8—N1	1.4054 (18)	N1—H1	0.8837
C8—O3	1.4892 (18)	O1—H1A	0.8551
C8—C9	1.5037 (19)		
			
O2—C1—O1	122.93 (13)	C14—C9—C10	120.58 (13)
O2—C1—C2	118.54 (13)	C14—C9—C8	109.02 (12)
O1—C1—C2	118.52 (12)	C10—C9—C8	130.40 (13)
C7—C2—C3	118.18 (12)	C9—C10—C11	117.69 (15)
C7—C2—C1	120.29 (13)	C9—C10—H10	121.2
C3—C2—C1	121.49 (12)	C11—C10—H10	121.2
C4—C3—C2	121.09 (13)	C10—C11—C12	121.48 (15)
C4—C3—H3	119.5	C10—C11—H11	119.3
C2—C3—H3	119.5	C12—C11—H11	119.3
C3—C4—C5	120.32 (12)	C13—C12—C11	120.85 (14)
C3—C4—H4	119.8	C13—C12—H12	119.6
C5—C4—H4	119.8	C11—C12—H12	119.6
N1—C5—C4	119.29 (12)	C12—C13—C14	117.30 (15)
N1—C5—C6	121.97 (12)	C12—C13—H13	121.3
C4—C5—C6	118.73 (12)	C14—C13—H13	121.3
C7—C6—C5	120.26 (13)	C9—C14—C13	122.06 (14)
C7—C6—H6	119.9	C9—C14—C15	108.59 (12)
C5—C6—H6	119.9	C13—C14—C15	129.34 (14)
C6—C7—C2	121.32 (13)	O4—C15—O3	121.22 (13)
C6—C7—H7	119.3	O4—C15—C14	129.98 (13)
C2—C7—H7	119.3	O3—C15—C14	108.80 (12)
N1—C8—O3	111.92 (11)	C5—N1—C8	122.57 (12)
N1—C8—C9	114.49 (12)	C5—N1—H1	117.3
O3—C8—C9	102.61 (11)	C8—N1—H1	117.7
N1—C8—H8	109.2	C1—O1—H1A	114.1
O3—C8—H8	109.2	C15—O3—C8	110.77 (11)
C9—C8—H8	109.2		
			
O2—C1—C2—C7	−0.4 (2)	C10—C11—C12—C13	1.7 (3)
O1—C1—C2—C7	−179.08 (14)	C11—C12—C13—C14	−0.9 (2)
O2—C1—C2—C3	177.30 (14)	C10—C9—C14—C13	1.9 (2)
O1—C1—C2—C3	−1.3 (2)	C8—C9—C14—C13	−177.47 (13)
C7—C2—C3—C4	2.5 (2)	C10—C9—C14—C15	−176.90 (13)
C1—C2—C3—C4	−175.24 (13)	C8—C9—C14—C15	3.73 (15)
C2—C3—C4—C5	−0.5 (2)	C12—C13—C14—C9	−0.8 (2)
C3—C4—C5—N1	176.53 (13)	C12—C13—C14—C15	177.69 (14)
C3—C4—C5—C6	−2.1 (2)	C9—C14—C15—O4	178.44 (15)
N1—C5—C6—C7	−175.90 (14)	C13—C14—C15—O4	−0.2 (3)
C4—C5—C6—C7	2.7 (2)	C9—C14—C15—O3	−1.20 (16)
C5—C6—C7—C2	−0.7 (2)	C13—C14—C15—O3	−179.88 (14)
C3—C2—C7—C6	−2.0 (2)	C4—C5—N1—C8	−178.74 (12)
C1—C2—C7—C6	175.86 (13)	C6—C5—N1—C8	−0.2 (2)
N1—C8—C9—C14	−126.09 (13)	O3—C8—N1—C5	63.86 (17)
O3—C8—C9—C14	−4.61 (14)	C9—C8—N1—C5	−179.92 (12)
N1—C8—C9—C10	54.6 (2)	O4—C15—O3—C8	178.43 (13)
O3—C8—C9—C10	176.11 (14)	C14—C15—O3—C8	−1.89 (15)
C14—C9—C10—C11	−1.1 (2)	N1—C8—O3—C15	127.16 (12)
C8—C9—C10—C11	178.09 (14)	C9—C8—O3—C15	3.93 (14)
C9—C10—C11—C12	−0.6 (3)		

### Hydrogen-bond geometry (Å, °)

**Table:**

*D*—H···*A*	*D*—H	H···*A*	*D*···*A*	*D*—H···*A*
N1—H1···O4^i^	0.88	2.11	2.9802 (17)	168
O1—H1A···O2^ii^	0.86	1.79	2.6438 (16)	175

Symmetry codes: (i) *x*−1, *y*, *z*; (ii) −*x*+1, −*y*+3, −*z*.
